# Revisiting X-linked agammaglobulinemia

**DOI:** 10.70962/jhi.20250020

**Published:** 2025-04-28

**Authors:** Hirokazu Kanegane, Kay Tanita, Madoka Nishimura, Dan Tomomasa, Kento Inoue, Toru Kanamori, Akira Nishimura, Hans D. Ochs

**Affiliations:** 1 Department of Child Health and Development, Graduate School of Medical and Dental Sciences, Institute of Science Tokyo, Tokyo, Japan; 2 Department of Pediatrics and Developmental Biology, Graduate School of Medical and Dental Sciences, Institute of Science Tokyo, Tokyo, Japan; 3 Department of Pediatrics, Graduate School of Medical Sciences, Kumamoto University, Kumamoto, Japan; 4 Department of Pediatrics, University of Washington, and Seattle Children’s Research Institute, Seattle, WA, USA

## Abstract

Discovered >70 years ago by Ogden Bruton, X-linked agammaglobulinemia (XLA), characterized by recurrent bacterial infections, hypo/agammaglobulinemia, and peripheral blood B-cell deficiency, is among the best-established inborn errors of immunity (IEIs) and one of the most well-documented single types of IEIs, the incidence of which is estimated to be between 1:100,000 and 1:200,000. However, although the pathogenesis of XLA is well understood, several issues remain open for discussion. In this review, we describe several unresolved issues, including noncoding *BTK* variants, contiguous deletion syndrome, *Helicobacter* infection, noninfectious neurodegeneration, renal involvement, and malignancies. The primary treatment for XLA, immunoglobulin replacement therapy, administered either intravenously or subcutaneously, has remained unchanged since its discovery. Allogeneic hematopoietic cell transplantation has been successful in some XLA patients, but there are still few reports. However, it may be considered as a treatment option in the future. Given that XLA is one of the most common types of IEIs, resolving these issues is a priority.

## Teaser

Although the pathogenesis of XLA is well understood, several issues remain open for discussion. We describe several unresolved issues, including non-coding *BTK* variants, contiguous deletion syndrome, Helicobacter infection, non-infectious neurodegeneration, renal involvement, and malignancies.

## Introduction

X-linked agammaglobulinemia (XLA) is characterized by recurrent bacterial infections starting in early childhood, diminished numbers of peripheral blood B cells, and reduced levels of serum immunoglobulin. XLA is among the best-established primary immunodeficiency diseases also known as inborn errors of immunity (IEIs), and it serves as the prototype of predominantly antibody deficiencies. Since the discovery of the disease in 1952 (1), immunoglobulin supplementation has been used as the primary treatment. The causative gene was identified in 1993 (2, 3). Although the clinical characteristics of XLA have been extensively studied, and most affected individuals survive into adulthood with adequate immunoglobulin replacement therapy (IgRT), some patients experience fatal complications and numerous issues associated with XLA remain unresolved. In this review, we describe important unmet challenges associated with XLA.

## The history of XLA

The first case of agammaglobulinemia, an 8-year-old boy who had experienced recurrent bacterial infections since age 4, was reported in 1952 by the American pediatrician Ogden C. Bruton at the Walter Reed Army Hospital (1). Whereas in normal individuals, serum electrophoresis identifies the different fractions of albumin and globulins (α1-, α2-, β-, and γ-globulin), the patient serum was found to lack the γ-globulin fraction (normal range: 10.5–20.3% of serum protein), recognized by Bruton as agammaglobulinemia. Based on this finding, Bruton decided to administer Cohn fraction II obtained from a pool of plasma collected from healthy donors. In response to these subcutaneous injections, the patient’s serum γ-globulin fraction increased to 4.6%, and he became free from recurrent infections. The observation confirmed that the γ-globulin fraction, later identified as immunoglobulin G, contains protective antibody with antibacterial properties. Similar reported cases of agammaglobulinemia in males provided evidence that the disease is X-linked, and, consequently, was named XLA. Although ataxia–telangiectasia (4), Wiskott–Aldrich syndrome (5), and severe congenital neutropenia (6, 7) were identified prior to the discovery of XLA, the study by Bruton is considered historically important, in that it describes the first laboratory diagnosis and treatment of an IEI.

More than 40 years after the discovery of XLA, its causative gene was discovered in 1993 by two independent groups. Tsukada et al. (2) identified a novel cytoplasmic tyrosine kinase, termed B-cell progenitor kinase (*Bpk*), expressed in B cells and myeloid cells of mice. Given its role in B-cell development, *BPK* was considered a candidate gene for XLA and was confirmed as the causative gene. Vetrie et al. (3) used positional cloning to identify the causative gene of XLA, a member of the Src family of proto-oncogenes, encoding a protein-tyrosine kinase, which was named agammaglobulinemia tyrosine kinase (*ATK*). *BPK* and *ATK* were subsequently recognized to have the same chromosomal location (Xq21.3-Xq22), similar structure (pleckstrin-homology, Tec homology, and three Src homology domains), and the same function in B-cell development. Consequently, it was agreed to rename the gene *Bruton tyrosine kinase* (*BTK*) in honor of Ogden Bruton, the discoverer of XLA (8, 9).

## Noncoding gene variants in *BTK*

Numerous pathogenic variants of *BTK* have been reported to date. As of February 2025, >2,400 variants have been submitted to the Leiden Open Variation Database, an open-source database developed by the Leiden University Medical Center in the Netherlands (https://databases.lovd.nl/shared/genes/BTK). *BTK* abnormalities reported (10, 11, 12, 13, 14, 15) include missense variants (20–37%), deletions (17–38%), nonsense variants (12–23%), splice site variants (0–20%), and insertions (3–8%), which mostly occur in the sequence downstream of exon 1 (correspond to exon 2 in the old nomenclature), which marks the start of the coding sequence. In contrast, variants in the 5′-UTR preceding exon 1 account for only 1% of all known variants (14). In this review, we discuss variants in the noncoding regions of *BTK* identified in patients with XLA.

PU.1 and Sp1 binding sites have been identified in the promoter region of the BTK promoter region, and mutants in this region have been found to suppress BTK transcription (16, 17). Furthermore, a variant in the promoter region (5′-UTR-58G>A) has been identified in an XLA patient (18), which was found to modify the invariable core sequence in the PU.1 binding site, thereby reducing binding to PU.1 and thus promoter activity. López-Granados (13) identified a four-base pair (AAAG) deletion in the PU.1 binding region, resulting in decreased BTK transcription and detectable BTK expression by flow cytometry. Another patient with a single base variant (c.-193A>G) presented with neutropenia and a mild clinical phenotype, with B cells representing 1% of all lymphocytes and IgG levels ranging from 300 to 450 mg/dl (19). However, mRNA expression was undetectable in monocytes and intracellular BTK expression was reduced in the patient. In 2021, six cases of agammaglobulinemia due to the variant in *SPI1*, which encodes the transcription factor PU.1, have been reported (20). A total of 22 agammaglobulinemic patients with PU.1-mutated agammaglobulinemia (PU.MA) have been identified (20, 21, 22), who are characterized by B-cell deficiency, as well as conventional and plasmacytoid dendritic cell deficiencies. Variants in the promoter region should be considered when XLA is clinically suspected, even if few B cells are present or BTK expression is detectable. In addition, a detailed investigation of XLA cases due to promoter variants may provide insight into other genetic causes of agammaglobulinemia.

In several studies, *BTK* variants have been identified downstream of exon −1 and in intron −1. Among these, a study conducted in the Netherlands identified a variant in the splice donor site of exon −1 (c.-31G>A), which resulted in aberrant splice products, leading to BTK deficiency (23). However, variants located a few bases distant from this site in exon −1 may cause abnormalities in transcriptional regulation (24, 25, 26). When *BTK* constructs harboring variants in both the promoter region and introns were inserted into a luciferase expression vector to assess transcriptional activity, the insertion of the (normal) promoter region alone induced a significant increase in transcriptional activity and the (normal) splice donor site equivalent to the variants (observed in one patient [23]) contributed to further enhancing transcription; however, when the variants observed in patients affecting either the promoter or intron −1 were inserted, almost no transcriptional activity occurred (24, 25, 26). Thus, although the detailed mechanisms remain unclear, it has been speculated that variants in intron −1 may lead to abnormal transcriptional regulation.

XLA has also occurred because of splice abnormalities caused by deep intronic variants. In one such case, the patient harbored a c.840-272G>T variant, which generated a splice donor site, leading to a splice abnormality between exons 8 and 9 (27). Although normal *BTK* c.DNA was observed, low-level BTK expression was detected in monocytes via intracellular flow cytometry. Deep intron variants have previously been identified in other types of IEIs, including NEMO deficiency (28, 29) and familial hemophagocytic lymphohistiocytosis type 3 (30, 31), the underlying mechanisms of which have been analyzed in detail. Splice variants can either generate new acceptor or donor sites that are recognized by the splicing complex, or, conversely, prevent recognition, thereby inhibiting normal mRNA production. In silico analysis tools, such as Splice AI software (https://spliceailookup.broadinstitute.org/#variant=X-101359619%20C-A&hg=38&distance=500&mask=1&ra=0), may contribute to identifying such pathogenic variants.

Whole-exome sequencing is a useful tool to analyze the exonic and nearby intronic regions of all genes. Although this approach covers <2% of the entire genome, it includes 85% of known disease-related gene variants and is a widely used diagnostic tool (32). Targeted gene panel next-generation sequencing is also a useful approach. However, as highlighted by the studies discussed herein, these methods may miss variants in noncoding regions or deep introns. To address these deficiencies, the utility of alternative techniques such as whole-genome and long-read sequencing potentially can enhance the diagnostic rates for XLA and other IEIs.

## XLA and chromosomal abnormalities

The translocase of the inner mitochondrial membrane 8 homolog A (*TIMM8A*) gene, located 770 bp centromerically from *BTK*, consists of two exons that function as a translocase in the inner mitochondrial membrane (33). Variants in *TIMM8A* have been associated with Mohr–Tranebjaerg syndrome (MTS), a condition characterized by progressive neurodegeneration, including sensorineural hearing impairment, dystonia, and motor difficulties. Given the close proximity of *TIMM8A* to *BTK*, a deletion encompassing both genes has been observed to result in XLA-MTS, a contiguous deletion syndrome first reported in a single family in 1996, and has since been identified in >20 patients (34, 35, 36, 37, 38, 39, 40, 41, 42, 43, 44, 45).

Deletions can also extend to the adjacent genes *TAF7L* and *DRP2* centromeric to *BTK*, with the largest reported deletion from *BTK* to *DRP2* being 196 kb (40). Diagnosis in these cases is often based on targeted polymerase chain reactions for *BTK*, *TIMM8A*, *TAF7L*, and *DRP2*; other methods include array comparative genomic hybridization, fluorescence in situ hybridization, and multiplex ligation-dependent probe amplification. Next-generation sequencing can also be used to identify chromosomal breakpoints (43, 45).

Clinically, patients with XLA-MTS experience recurrent infections considered to be a consequence of XLA, and symptoms of progressive neurodegeneration due to MTS. Most affected patients initially present with frequent recurrent infections during infancy, followed by symptoms of MTS, with ∼80% of these patients exhibiting hearing impairment, along with speech delay at around 3 years of age (Table 1) (34, 35, 36, 37, 38, 39, 40, 41, 42, 43, 44, 45). However, XLA alone is occasionally comorbid with hearing loss caused by recurrent otitis media. XLA-MTS is typically characterized by progressive sensorineural hearing loss. In addition, dystonia and psychomotor retardation may develop prior to school age, whereas visual impairment, intellectual disability, and dysgraphia typically develop after 15 years of age. The associated neurologic manifestations tend to be diverse and unrelated to the extent of gene deletion.

**Table 1.** Clinical characteristics of MTS in 23 patients with XLA-MTS

Symptoms	Number of patients	Median onset (years)	References
Hearing impairment	18 (78%)	3.5	(33, 34, 35, 36, 37, 38, 39, 40, 41, 42, 43, 44, 45)
Speech delay/abnormality	12 (52%)	3	(35, 37, 38, 39, 42)
Dystonia	5 (22%)	5	(34, 36, 37, 44)
Psychological developmental disorder	5 (22%)	7	(34, 37, 40, 42, 45)
Motor developmental delay	3 (13%)	3	(37, 42)
Visual impairment	3 (13%)	15	(34, 36, 41)
Intellectual disability	2 (9%)	17.5	(34, 36)
Dysgraphia	2 (9%)	17.5	(36, 45)

XLA, X-linked agammaglobulinemia; MTS, Mohr–Tranebjaerg syndrome. Median onset is shown as years.

In a single case, XLA was found to be associated with Klinefelter syndrome (46), with both X chromosomes of the patient having the same *BTK* variant as those observed in his mother, thereby tending to indicate nonsegregation of the X chromosomes during the second meiotic division in oogenesis.

## *Helicobacter* infection in XLA

Bacteria in the genus *Helicobacter* are notable pathogens detected in patients with XLA. They are classified as either *Helicobacter pylor*i or non-*Helicobacter pylori Helicobacter* (NHPH); the latter include enterohepatic *Helicobacter* species such as *Helicobacter cinaedi*, *Helicobacter fenneliae*, *Helicobacter canis*, and *Helicobacter bilis. H*. *pylor*i have been established to be the causative agent of gastritis. In XLA patients, the mucosal production of IgA and IgG is disrupted, resulting in a defective immune defense system in the gut (47). Similarly, selective IgA deficiency exacerbates uncontrolled progression of atrophic gastritis because of *H*. *pylor*i infection (48). Solid tumors in patients with XLA occur primarily in the gastrointestinal tract and are often associated with chronic *H*. *pylor*i infections (49). For these reasons, patients with XLA should be rigorously screened by culture and by PCR for *H*. *pylor*i infection (50), as antibody tests will yield false-negative results.

Enterohepatic NHPH species have also been identified as pathogens in patients with XLA, often associated with cellulitis and pyoderma gangrenosum (51, 52, 53, 54, 55, 56, 57, 58, 59, 60, 61, 62, 63, 64, 65, 66, 67, 68, 69). *H. cinaedi* and *H. bilis* have been reported to occasionally be the primary pathogens causing skin ulcers in XLA patients. Detecting NHPH in skin lesions is challenging, requiring long-term blood culture and the application of supplementary microbiological methods, such as PCR using specific primers for 16S ribosomal RNA and mass spectrometry. Patients with NHPH skin infections require antimicrobial treatment for at least 6 wk (57). IgRT alone is ineffective in preventing NHPH infections (57). Accordingly, chronic skin and soft tissue infections in patients with XLA should be investigated for NHPH employing long-term blood cultures.

## Noninfectious progressive neurodegeneration in XLA

Patients with XLA are often associated with central nervous system (CNS) infections, most commonly viral encephalitis and bacterial meningitis. However, progressive neurodegeneration without identifiable infection has been observed in patients with XLA and other IEIs (70). A total of 15 patients with XLA associated with neurodegeneration have been reported to date (70, 71, 72, 73, 74, 75, 76, 77, 78, 79). The mean age at diagnosis of XLA was 3.25 years, and the mean age at the onset of neurodegeneration was 10.15 years. Thus, the average time from the diagnosis of XLA to the onset of neurodegeneration was ∼7 years; however, two patients were diagnosed with XLA and neurodegeneration simultaneously. Thirteen patients had received IgRT before the onset of the neurologic symptoms. The outcome of this complication was poor, being fatal in most patients (10/13). A 15-year-old XLA patient with fatal encephalitis was identified using unbiased pyrosequencing as being due to astrovirus infection (80). Neurodegeneration in XLA patients may be caused by viral or other infectious agents that may have not been identified. BTK is expressed not only in B cells but also in innate cells, including microglia in the CNS, inhibitor of which have been studied in mouse models for multiple sclerosis (81). It is speculated that microglia may play important roles in neuroinflammation and that impaired microglial function may influence the development of neurodegeneration in patients with XLA.

## Renal involvement in XLA

Kidney disease, particularly nephritis, is a rare complication of XLA (82, 83, 84, 85, 86, 87, 88, 89, 90, 91, 92). The clinical presentations of nephritis in XLA patients are summarized in Table 2. In most cases of XLA-related nephritis, the infections were controlled in response to intravenous immunoglobulin (IVIG) treatment, or the nephritis occurred in the absence of major infections.

**Table 2.** Clinical characteristics of XLA patients presenting with nephritis

Patient	Age at the diagnosis of XLA (years)	Age at the diagnosis of kidney disease (years)	*BTK* variant	Proteinuria (g/gCre)	Hematuria	Serum creatinine (mg/dl)	LM	IF	Histological diagnosis	Treatment	Prognosis	References
1	50	47	c.1908 + 1G>A	0.55	3+	1.0	Mesangial proliferation	IgA (+)	IgAN	ACE-I	Improvement of proteinuria	(83)
2	1	8	c.1379T>G, p.Leu460Trp	0.5	3+	0.34	Mesangial proliferation/endocapillary proliferation	IgA (+), C3 (+), Fib (+)	IgAN	PSL	Improvement of proteinuria	(83)
3	0.3	10	c.787del, p.Tyr263Thrfs*14	±	2+	0.4	Mesangial proliferation/duplication of the basement membrane	IgG (+), C3 (+)	MPGN	MPT + PSL	Improvement of proteinuria	(83)
4	0.2	3	c.612insA	0.86	3+	0.2	Mesangial proliferation/duplication of the basement membrane	IgG (+), C3 (+)	MPGN	MPT + PSL	Improvement of proteinuria	(83)
5	0.9	23	Exon 6–18 duplication	0.15	3–10/HPF	1.1	Deposition of immune complex in the basement membrane	IgG (+)	MN	None	Spontaneous remission	(83)
6	5	6	c.347C>T, p.Pro116Leu	0.34	3+	0.4	Cellular proliferation in mesangial/endocapillary electronic dense deposits	IgG (+), IgA (+), C3 (+), C4 (+), IgM (+), C1q (+)	Immune complex glomerulonephritis	PSL + MMF	Improvement of proteinuria	(83)
7	0.6	10	p.Arg288Gln	0.63	>50/HPF	0.3	Mesangial proliferation/duplication of the basement membrane	IgG (3+), C3 (2+), C4 (+), C1q (2+)	MPGN	ACE-I/change of IVIG preparation	Improvement of proteinuria	(83)
8	11	11	p.Tyr40Cys	+++	4/HPF	1.29	Mesangial proliferation/fibrocellular crescent formation	C3 (+++)	Mesangial proliferative nephritis	Antibiotics and IVIG for respiratory infection	Improvement of proteinuria	(88)
9	11	12	p.Tyr40Cys	++	30/HPF	Normal range	Mesangial proliferation/duplication of the basement membrane	C 3(+), IgG (+)	MPGN	Antibiotics and IVIG for respiratory infection	Improvement of proteinuria	(88)
10	6	6	c.240G > A	+	970/HPF	0.42	Mesangial proliferation	IgA (+++), C3 (++), IgM (+)	IgAN	MPT + PSL + MMF	Improvement of proteinuria	(89)
11	7	12	c.1361delA	3	150/HPF	0.78	Mesangial proliferation/duplication of the basement membrane	C3 (+), IgG (+)	MPGN	MPT + PSL	Improvement of proteinuria	(90)
12	0.25	20	p.Gln412*	0.14	14-/HPF	1.5	Tubulointerstitial cell infiltrate/crescent formation	IgG (+)	TIN	Change of IVIG preparation /MPT/PSL	Improvement of proteinuria/CKD stage 3	(83)
13	0.5	11	Exon 6–7 duplication	0.26	3+	N/A	Tubulointerstitial cell infiltrate/crescent formation	IgG (+), C3 (+)	TIN	Change of IVIG preparation /MPT	ESRD	(83)

XLA, X-linked agammaglobulinemia; *BTK*, *Bruton tyrosine kinase*; LM, light microscopy; IF, immunofluorescence; HPF, high-power field; N/A, not accessed; Fib, fibrinogen; IgAN, IgA nephritis; MPGN, membranoproliferative glomerulonephritis; MN, membranous nephropathy; TIN, tubulointerstitial nephritis; ACE-I, angiotensin converting enzyme inhibitor; MPT, methylprednisolone pulse therapy; PSL, prednisolone; MMF, mycophenolate mofetil; IVIG, intravenous immunoglobulin; CKD, chronic kidney disease; ESRD, end-stage renal disease.

Based on the histological findings of renal biopsies, XLA-related nephritis can be classified into chronic glomerulonephritis (CGN) and tubulointerstitial nephritis (TIN), which are characterized by different features, particularly with respect to immune phenotypes (83, 84, 85, 86, 87, 88, 89, 90, 91, 92). Patients in the CGN group generally have atypical/mild XLA profiles. Pathological features of renal biopsies taken from this CGN group are characterized as glomerulonephritis with mesangial proliferation and immune complex deposition. On the basis of these immunological and pathological findings, it has been hypothesized that leaky pathogenic B cells may produce antibodies that could play a role in the formation of immune complexes, causing immune-mediated glomerulonephritis in these CGN patients (83).

In contrast to patients assigned to the CGN group, those with the TIN phenotype typically have immunologic profiles that are characteristic of classic XLA. Most patients with TIN have a history of >10 years of IVIG treatment prior to the onset of nephritis, which might indicate that TIN develops as a consequence of antibody-independent mechanisms (83). Considering that BTK mediates signaling through Toll-like receptor 9, other antibody-independent or BTK-dependent mechanisms may cause the TIN phenotype and other autoimmune and inflammatory manifestations of XLA. Interestingly, the results of immunohistochemical staining in the TIN group demonstrated remarkable tissue infiltration of CD8^+^ T cells and deposition of IgG. These features are also observed in cases of lupus-related TIN or acute kidney graft rejection in which cellular immunity and immune complexes are involved. The absence of B-cell infiltration and follicular-like structures, as observed in lupus-related TIN cases, and the low serum IgG levels characteristically seen in the TIN group raise the possibility that transient high IgG concentration, due to exogenous IVIG treatment, causes the formation of immune complexes.

Notably, the patients in these two groups tend to differ in terms of treatment modality and prognosis of XLA-related nephritis. Patients in the CGN group have less severe nephritis, and treatment with steroids and immunosuppressants is recommended. Some patients in this group have done well on conservative treatment. Compared with patients in the CGN group who are likely to recover without prolonged kidney dysfunction or proteinuria, those in the TIN group tend to have a less favorable course.

As an alternative to IVIG, subcutaneous immunoglobulin (SCIG) therapy avoids intermittently high serum IgG concentrations (93). If immune complexes are indeed generated by transiently high levels of serum IgG resulting from large quantities of IVIG, a switch to a SCIG preparation designed for weekly infusions would less likely serve as a source of immune complexes detected in the kidneys of CGN patients (83). At present, however, there have been an insufficient number of case studies that have evaluated the preventive effect of SCIG on nephritis.

## XLA and malignancies

In addition to diverse infections, patients with XLA are susceptible to developing malignancies, as reported in two large cohort studies (94, 95). Table 3 lists the details of individual XLA patients who had developed solid tumors, which often occurred in early life (94, 95, 96, 97, 98, 99, 100, 101, 102, 103, 104, 105, 106, 107, 108, 109), with more than half consisting of upper and lower gastrointestinal adenocarcinomas. Patients with XLA are susceptible to chronic infections with gastrointestinal parasites and bacteria, including *Giardia lamblia*, *Campylobacter jejuni*, and *H. pylori* (110); the chronic inflammation associated with these pathogens is believed to contribute to the pathogenesis of gastrointestinal malignancies (111). Gastrointestinal tumors also occur frequently via the same mechanism in patients with common variable immunodeficiency who often present with chronic gastrointestinal inflammation (112). Upper gastrointestinal endoscopy, colonoscopy and serum pepsinogen testing are helpful to monitor gastrointestinal tumors, although none of these methods has proven to be superior (49, 113). Endoscopy is the most effective approach to detect gastrointestinal tumors in XLA patients with digestive symptoms. Considering that all but 2 of the 15 XLA patients listed in Table 3 were <40 (8 were 30 or less) years of age when diagnosed with gastric or colorectal cancer, it has been recommended to start routine endoscopic screening at a younger age than recommended for the general population (107). At present, there are no specific management protocols recommended for the treatment of gastrointestinal tumors in patients with XLA, nor is there evidence to indicate that the use of IgRT for infection control prevents gastrointestinal tumors.

**Table 3.** Solid tumors in XLA patients

Types of solid tumor	Age at cancer diagnosis (years)	Outcome	References
Gastric adenocarcinoma	23	Alive	(104)
Gastric adenocarcinoma	26	ND	(104)
Gastric adenocarcinoma	25	Dead	(100)
Gastric adenocarcinoma	15	Alive	(101, 102)
Gastric adenocarcinoma	45	ND	(105)
Gastric adenocarcinoma	30	Dead	(49)
Gastric carcinoma	37	Dead	(93)
Colorectal cancer	30	Dead	(106)
Colorectal cancer	36	Dead	(96)
Colorectal cancer	ND	ND	(108)
Colorectal cancer	22	Alive	(95)
Colorectal cancer	37	Alive	(98)
Colorectal cancer	45	Alive	
Colon adenocarcinoma	33	Alive	(93)
Colon adenocarcinoma	34	Dead	(93)
Colon adenocarcinoma	ND	ND	(94)
Colon neuroendocrine carcinoma	10	Dead	(104)
Lung squamous cell carcinoma	32	Dead	(98)
Liver cancer	7	Dead	(106)
Liver carcinoma with HCV	38	Dead	(93)
Ependymal astrocytoma variant giant cells	11	Alive	
Papillary thyroid carcinoma, follicular type	36	Alive	
Osteosarcoma	ND	ND	(94)
Reticulum cell sarcoma	ND	ND	

HCV, hepatitis C virus; ND, no data.

Patients with XLA can also develop hematologic malignancies, with higher prevalence of acute leukemia in XLA patients than in the general population (114). Table 4 presents details of hematologic malignancies observed in patients with XLA, who had developed acute lymphoblastic leukemia (ALL), acute myeloid leukemia (AML), non-Hodgkin lymphoma, and T-cell lymphoma (95, 114, 115, 116, 117, 118, 119, 120, 121, 122, 123, 124). To clarify the somatic molecular abnormalities presenting in hematologic malignancies associated with XLA, several investigators performed cytogenetic and genomic analyses of B-ALL and AML in XLA. These studies identified somatic loss-of-function variants in the tumor suppressors *CDKN2A* and *TP53* and monosomy 7 in all patients evaluated. Leukemogenesis follows the two-hit theory, highlighting the importance of maturation arrest and genetic alterations, while providing survival or proliferation advantages. The arrest of B-cell and myeloid cell differentiation caused by germline *BTK* variants represents a first hit, which, when combined with somatic tumor suppressor gene alterations (second hit), may lead to acute leukemias (114). The findings that *Btk/p53*-deficient mice undergo heightened proliferation of immature B cells (pro/pre-B cells and late pre-B cells) support this hypothesis (125). However, the mechanism underlying the tumorigenesis of lymphomas in XLA remains to be clarified. XLA patients with hematologic malignancies should receive the standard treatment protocols used for de novo cases. There is no clear evidence to suggest that XLA patients with hematologic malignancies should routinely undergo allogeneic hematopoietic stem cell transplantation (HSCT), not only to cure leukemia, but also for immunological recovery. HSCT may, nevertheless, be indicated for the treatment of hematopoietic malignancies, recurrent or life-threatening infections, or other uncontrollable severe complications (126). At present, however, there are limited data available regarding the prognosis of hematologic malignancies in patients with XLA.

**Table 4.** Hematologic malignancies in XLA patients

Types of hematologic malignancy	HSCT	Age at cancer diagnosis (years)	Outcome	References
Lymphoma	ND	ND	ND	(119)
Lymphoma	ND	ND	ND	(95)
AML	PBSCT	13	Alive	(115)
T-LGLL	No	47	Alive	(116)
CD8^+^ granulomatous cutaneous T-cell lymphoma	ND	33	Alive	(117)
Primary cutaneous peripheral T-cell lymphoma	No	28	Alive	(122)
CD8^+^ T-cell lymphoma	PBSCT	24	Alive	(121)
ALL	No	3	Dead	(124)
NHL	No	12	Alive	(118)
BCP-ALL	No	10	Alive	(114, 120)
BCP-ALL	PBSCT	24	Alive	(114, 123)
AMKL	No	9 mo	Alive	(114)

HSCT, hematopoietic stem cell transplantation; ND, no data; AML, acute myeloid leukemia; PBSCT, peripheral blood stem cell transplantation; T-LGLL, T-cell large granular lymphocytic leukemia; ALL, acute lymphoblastic leukemia; NHL, non-Hodgkin lymphoma; BCP, B-cell precursor; AMKL, acute megakaryoblastic leukemia.

## Treatment for XLA

Fundamental treatments for XLA include therapeutic and prophylactic antibiotic administration and IgRT, which was initiated in 1952 by Bruton (1). Although initially administered subcutaneously, the intramuscular delivery of IgRT became prevalent in the 1960s. However, intramuscular injections are painful, while the amount of IgG tolerated by the patient is restricted, resulting in low serum IgG levels and poor infection control. In the 1970s, IgG preparations were developed for intravenous infusion, allowing high-dose administration of IgG, resulting in better infection control. In the early 1990s, SCIG reemerged in Scandinavia and the United Kingdom and its use became widespread in the 2000s. Currently, all patients with XLA are treated with IVIG or SCIG, and most survive into adulthood. However, some patients still develop severe or fatal infections.

Although IgRT is considered the standard therapy for XLA, some patients with severe infectious and noninfectious complications may require HSCT (95, 115, 127). HSCT has also been used to treat XLA patients in countries where IgRT is not available for economic reasons or lack of supply. An international survey of using HSCT as a cure for XLA based on the treatment of 22 patients with uncontrolled infections (16 patients), hematologic malignancies (3 patients), and other serious complications (3 patients) was recently completed (126). Different conditioning regimens were used, including myeloablative conditioning (MAC) (4/22, 18%), reduced-toxicity MAC (10/22, 45%), and reduced-intensity conditioning (RIC) (8/22, 36%). Engraftment was achieved in 95% of patients, with 2-year overall survival of 86% and event-free (events: primary graft failure, secondary graft failure, or death) survival rates of 77%. 95% of patients achieved complete (50%) or stable high-level mixed chimerism. Given that IgRT affects the quality of life of XLA patients, the proportion of patients able to discontinue IgRT 1 year after HSCT was determined and found to be 89%. HSCT for XLA requires a conditioning intensity that allows engraftment and stable donor chimerism, as, except for mature B cells and myeloid cells, most immune cells are unaffected. To minimize the risk of late complications, RIC regimens, such as fludarabine-based regimens, are recommended. Although IgRT continues to remain the gold standard treatment for XLA, considering that long-term IgRT is more expensive than HSCT when health status is defined by quality-adjusted life years (128), successful HSCT with subsequent IgRT discontinuation may improve quality of life and reduce the longtime costs for patients with XLA. In summary, at present, the safety of HSCT has yet to be thoroughly assessed and it is currently considered a salvage therapy for XLA patients with severe infections, hematologic malignancies, or other lethal complications. However, HSCT may be considered a relative indication in situations in which continued IgRT is difficult. The demonstrated efficacy of HSCT for XLA indicates that gene therapy may be as effective for XLA as for severe combined immunodeficiency and other IEIs and preclinical trials of gene therapy for XLA are currently underway (129).

## Conclusion

XLA is the best-characterized IEI in terms of the numbers of patients studied using evidence-based approaches to establish the diagnosis and design effective treatment. However, several issues relating to XLA remain unresolved and were addressed in this review, including *Helicobacter* infection, progressive neurodegeneration, renal disease, malignancy, and HSCT. In the foreword to the first edition of Primary Immunodeficiency Diseases, Robert A. Good stated that “studying XLA will continue to reveal fundamental issues in lymphology and immunobiology,” a prediction that remains true. Although XLA is rarely associated with autoimmune diseases, the pathogenesis and effective treatment of this condition have yet to be sufficiently established. While comparatively few patients undergo HSCT, IgRT remains the standard treatment for XLA, with the caveat that plasma-derived products pose limitations related to availability, safety, and cost (130), highlighting the necessity to develop viable alternatives to IgRT, with the possibility of curing the disease.
